# *Ectomyelois* Heinrich, 1956 in China, with descriptions of two new species and a key (Lepidoptera, Pyralidae, Phycitinae)

**DOI:** 10.3897/zookeys.559.6076

**Published:** 2016-02-03

**Authors:** Yingdang Ren, Linlin Yang

**Affiliations:** 1 Institution of Plant Protection, Henan Academy of Agricultural Sciences, Henan Key Laboratory of Crop Pest Control, Key Laboratory of Integrated Pest Management on Crops in Southern Region of North China, Zhengzhou 450002, China; 2 College of Life Sciences, Nankai University, Tianjin 300071, China

**Keywords:** Lepidoptera, Pyralidae, Phycitinae, Ectomyelois, Ectomyelois ceratoniae, new species, key, China

## Abstract

Only three species belonging to the genus *Ectomyelois* Heinrich, 1956 are recorded from China, of which two species, *Ectomyelois bipectinalis* **sp. n.** and *Ectomyelois furvivena* **sp. n.** are described as new. We discuss the status of *Ectomyelois* that has been treated as a junior synonym by previous authors; we treat it as a valid genus, **revised status**, based on characters of the venation and female genitalia. Photographs of the adults and illustrations of the genitalia are given, along with a key to the three known Chinese species.

## Introduction

*Ectomyelois* was established by Heinrich (1956) with *Myelois decolor* Zeller, 1881 as the type species. It is a small genus consisting of six species: *Ectomyelois ceratoniae* (Zeller, 1839), *Ectomyelois decolor* (Zeller, 1881), *Ectomyelois furvidorsella* (Ragonot, 1888), *Ectomyelois muriscis* (Dyar, 1914), *Ectomyelois zeteki* Heinrich, 1956, and *Ectomyelois austrella* Neunzig & Goodson, 1992. Most are Neotropical, but *Ectomyelois ceratoniae* also occurs in the Oriental region. All but one species was described in detail by Heinrich (1956). *Ectomyelois* was once treated as a junior synonym of *Spectrobates* Meyrick by Roesler (1968) and subsequently of *Apomyelois* Heinrich by Roesler & Küppers (1981). A few authors followed these treatments (e.g. Neunzig 1979; Roesler 1983; Heppner and Inoue 1992; Leraut 2002), but most authors (Munroe 1983; Inoue 1982; Palm 1986; Sinev 1986; Goater 1986; Neunzig 1990; Balinsky 1994; Yamanaka 2013) treated *Ectomyelois* as a valid genus. Indeed, there is little to separate *Ectomyelois* from *Apomyelois* in the male genitalia, but the two genera can be distinguished by the venation and the different place of inception of the ductus seminalis from the corpus bursae in the female genitalia. Here, we agree that *Ectomyelois*, revised status, does indeed represent a valid genus.

*Ectomyelois* was only represented by the common carob moth *Ectomyelois ceratoniae* in China before this study. Herein the three species are described, including two new species: *Ectomyelois bipectinalis* sp. n. and *Ectomyelois furvivena* sp. n.

## Material and methods

Genitalia dissections were carried out following the methods described by Li (2002). The photographs of the adults and venation were taken with a Leica M205A, and photographs of the genitalia and details of head were taken with a Leica DM750, with Leica Application Suite 4.2 software to capture images. All the specimens examined are deposited in NKUM unless otherwise noted.

### Abbreviations

BMNH
Natural History Museum, London, UK 


HAASM
 Insect Collection, Institution of Plant Protection, Henan Academy of Agricultural Sciences, Zhengzhou, China 


NKUM
 Insect Collection, College of Life Sciences, Nankai University, Tianjin, China 


USNM
 National Museum of Natural History, Smithsonian Institution, Washington, D.C. 20560, U.S.A. 


ZMHB
Museum für Naturkunde, Universität Humboldt, Invalidenstrasse 43, 104 Berlin, Germany 


TD
 Type depository 


TL
 Type locality 


## Systematic part

### Ectomyelois

Taxon classificationAnimaliaLepidopteraPyralidae

Heinrich, 1956

Ectomyelois Heinrich, 1956: 43. Type species: *Myelois decolor* Zeller, 1881, by original designation.

#### Diagnosis.

Antenna of male usually shortly ciliate (bipectinate in *Ectomyelois bipectinalis* sp. n.), basal shaft without notch or other modifications, of female simple. Labial palpus upturned, nearly reaching apex, third segment distinctly shorter than second. Forewing with R_2_ closely approximate to the stalk of R_3+4_+R_5_, M_2_ and M_3_ stalked for less than half of their length. Hindwing with Sc + R_1_ and Rs strongly anastomosed for most of their lengths, M_2_ and M_3_ stalked for not over half of their length. Male genitalia with uncus subtriangular to bell-shaped, apical projection of gnathos simple, slightly bent and furcated at apex, transtilla well developed, juxta U-shaped, with lateral lobes stout, vinculum U-shaped, more truncate and less tapering, phallus without cornutus. Female genitalia with signum consisting of an elongate patch of scobinations (absent in *Ectomyelois furvidorsella*) and ductus seminalis from corpus bursae near junction of corpus bursae and ductus bursae.

*Ectomyelois* is similar to *Apomyelois*, but can be distinguished from the latter by the forewing with R_2_ closely approximate to the stalk of R_3+4_+R_5_, and the female genitalia with the ductus seminalis arising from the corpus bursae near junction of the corpus bursae and ductus bursae. In *Apomyelois*, the forewing with R_2_ shortly stalked with R_3+4_+R_5_, and the female genitalia with the ductus seminalis arising from anterior end of the corpus bursae.

#### Distribution.

China (Gansu, Guangdong, Guangxi, Hainan, Taiwan, Yunnan), India, Sri Lanka, Sikkim, Israel, Mediterranean, Central Europe, Norway, United Kingdom, North Africa, Australia, Argentina, United States, Cuba, Haiti, Puerto Rico, Jamaica, Bahamas, Guatemala, Costa Rica, Colombia, Panama, Venezuela, Guiana, Surinam, Bolivia, Guyana, French Guiana, Brazil.

#### Key to species of *Ectomyelois* from China

**Table:** 

1	Forewing with narrow grayish white, distinctly notched antemedial line (Fig. 1)	***Ectomyelois ceratoniae***
–	Forewing with antemedial line invisible	**2**
2	Male flagellum bipectinate (Fig. 3b); transtilla trefoiled (Fig. 7); corpus bursae three times as long as wide (Fig. 11)	***Ectomyelois bipectinalis* sp. n.**
–	Male flagellum simple, not bipectinate (Fig. 5b); transtilla inverse-goblet shape (Fig. 9); corpus bursae twice as long as wide (Fig. 12)	***Ectomyelois furvivena* sp. n.**

**Figures 1–6.**
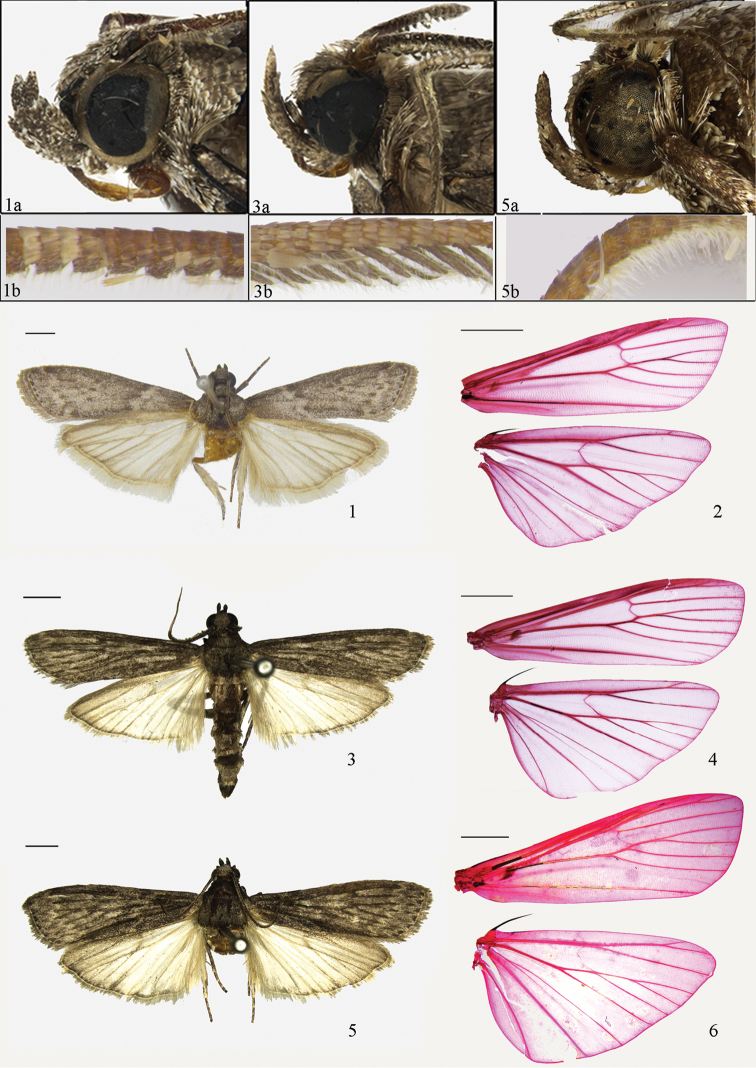
Adults of *Ectomyelois* spp. **1** *Ectomyelois ceratoniae*, male (1a, head; 1b, antenna) **2** *Ectomyelois ceratoniae*, venation, slide No. RYD04529w **3** *Ectomyelois bipectinalis* sp. n., paratype, male (**3a** head **3b** antenna) **4** *Ectomyelois bipectinalis* sp. n., venation, slide No. RYD04718w **5** *Ectomyelois furvivena* sp. n., paratype, male (**5a** head **5b** antenna) **6** *Ectomyelois furvivena* sp. n., venation, slide No. RYD04529w. Scale bars: 2.0 mm.

**Figures 7–9.**
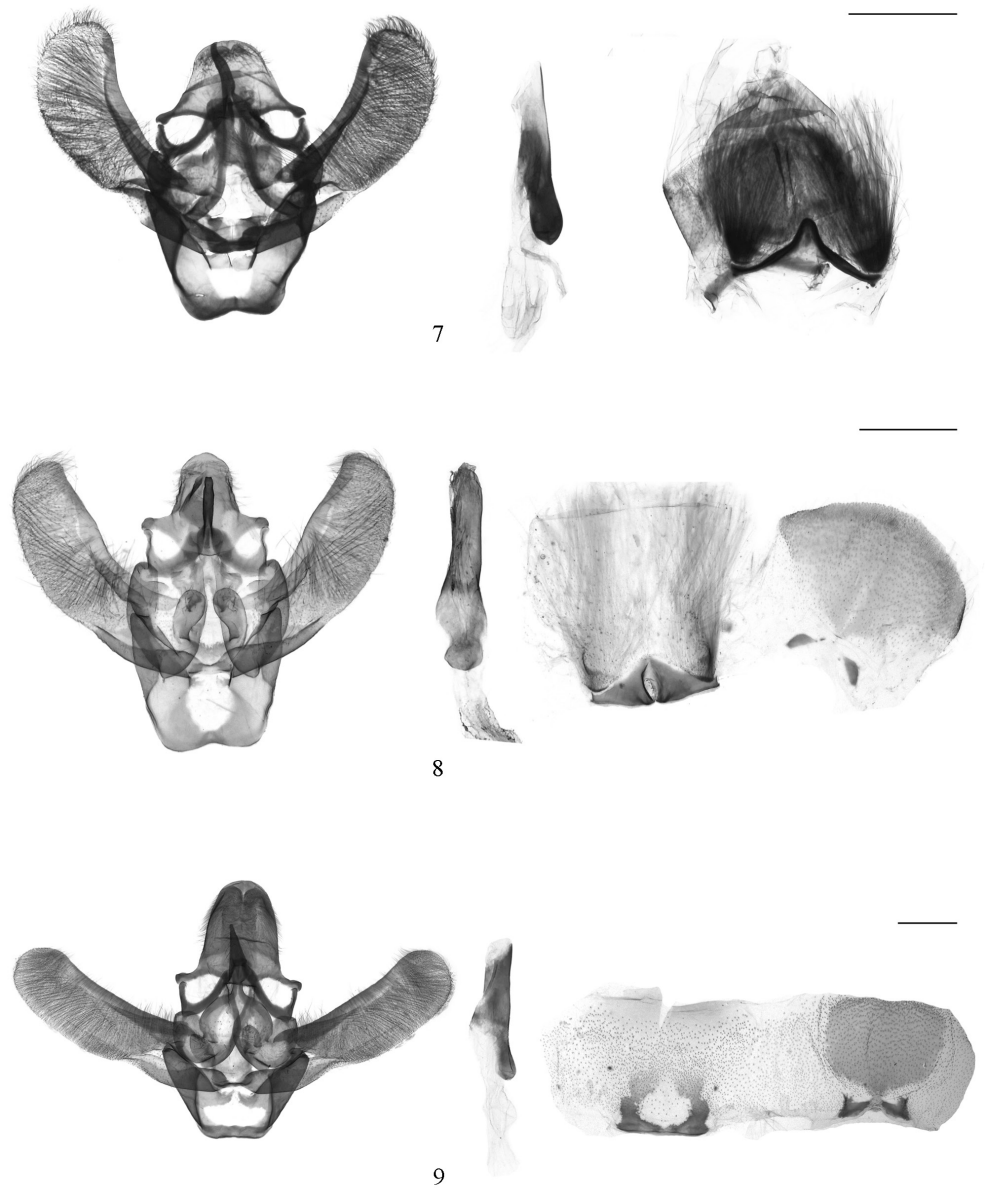
Male genitalia of *Ectomyelois* spp. **7** *Ectomyelois ceratoniae*, slide No. LJY10595 **8** *Ectomyelois bipectinalis* sp. n., paratype, slide No. RYD04718 **9** *Ectomyelois furvivena* sp. n., paratype, slide No. LHX14084. Scale bars: 0.5 mm.

### Ectomyelois ceratoniae

Taxon classificationAnimaliaLepidopteraPyralidae

(Zeller, 1839)

Figs 1 , 2 , 7 , 10

Myelois ceratoniae Zeller, 1839: 176. TL: Laibach, Austria. TD: BMNH.
Phycis ceratoniella Fischer von Röeslerstamm, 1839: 147. TL: Laibach, Austria. TD: unknown.
Trachonitis pryerella Vaughan, 1870: 130. TL: London, England. TD: BMNH.
Myelois tuerckheimiella Sorhagen, 1881: 103. TL: Berlin, Germany. TD: ZMHB.
Euzophera zellerella Sorhagen, 1881: 104. TL: Berlin, Germany. TD: unknown.
Phycita dentilinella Hampson, 1896: 91. TL: Manipur, India. TD: BMNH.
Hypsipyla psarella Hampson, 1903: 30. TL: Sikhim, India. TD: BMNH.
Heterographis rivularis Warren & Rothschild, 1905: 31. TL: Nakheila, Sudan. TD: unknown.
Myelois oporedestella Dyar, 1911: 30.TL: Florida, USA. TD: USNM.
Myelois phoenicis Durrant, 1915: 303. TL: Constantine, Algeria. TD: BMNM.
Laodamia durandi Lucas, 1950:142. TL: Tunisia. TD: unknown.
Apomyelois ceratoniae (Zeller): Roesler and Küppers 1981: 80.
Ectomyelois ceratoniae (Zeller): Heinrich 1956: 44.

#### Material examined.

**CHINA**: **Guangdong**: 9 ♂♂, 1 ♀, Mt. He (22°45'N, 112°57'E), 09−10-X-2002, coll. Guilin Liu & Binglan Zhang, gen. slide nos. RYD04529m, RYD04530f; 1 ♂, same data as former except dated 6-XI-2002. **Guangxi**: 1 ♀, Milv (21°59'N, 107°52'E), Nanping, Shangsi, 770 m, 3-IV-2002, coll. Shulian Hao & Huaijun Xue, gen. slide no. RYD04658; 1 ♂, Yachang Yard, Leye County (24°47'N, 106°33'E), 665 m, 24-VII-2004, coll. Jiasheng Xu, gen. slide no. KDH05263; 1 ♀, Longrui (22°45'N, 110°55'E), 18-VIII-2011, coll. Muchun Cheng, gen. slide no. RYD20120185 (deposited in HAASM); 1 ♂, 1 ♀, Nonggang (23°14'N, 108°10'E), 20-VIII-2011, coll. Dandan Zhang, gen. slide no. RYD2014237 (deposited in HAASM). **Hainan**: 1 ♂, Mt. Diaoluo (18°39'N, 109°54'E), 29-V-2007, 80 m, coll. Zhiwei Zhang & Weichun Li, gen. slide no. LJY10595. **Yunnan**: 1 ♂, 1 ♀, Ganlanba (22°45'N, 101°08'E), Xishuangbanna, 19-IV-1995, coll. Guangyun Yan, gen. slide nos. RYD04615m, LJY10107f; 1 ♂, 4 ♀♀, Mt. Yunpan (23°44'N, 100°39'E), Puer, 1600 m, 6-VII-2013, coll. Linlin Yang, gen. slide nos. RYD2014219m, RYD2014221f (deposited in HAASM).

#### Diagnosis.

**Figures 10–12.**
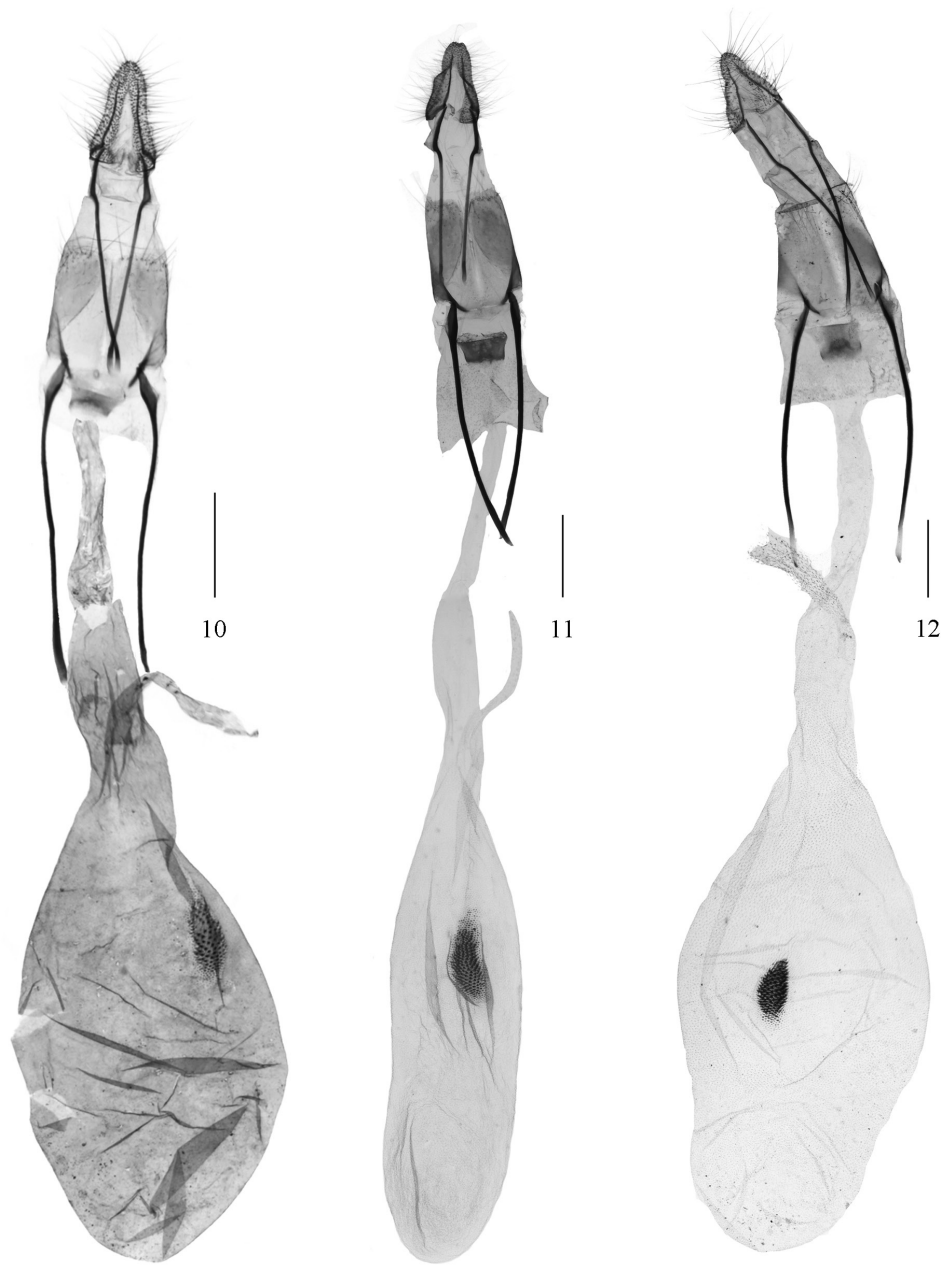
Female genitalia of *Ectomyelois* spp. **10** *Ectomyelois ceratoniae*, slide No. RYD04658 **11** *Ectomyelois bipectinalis* sp. n., paratype, slide No. LJY10107 **12** *Ectomyelois furvivena* sp. n., paratype, slide No. RYD04744. Scale bars: 0.5 mm.

Wingspan 15.0−22.00 mm (Fig. 1). *Ectomyelois ceratoniae* can be recognized by the following characters: the forewing with a narrow, distinctly notched antemedial line, the hindwing with free element of Sc+R_1_ very short (Fig. 2); the uncus is bell-shaped, basally protruded on both sides, the apical projection of gnathos is stout, gently curved, about same length of the uncus, the trefoiled transtilla includes a pair of inflated bases and a more constricted central projection, the basally rectangular juxta with a pair of stout lateral lobes in the male genitalia (Fig. 7); and the ovate corpus bursae with signum is an elongate patch of scobinations, the ductus seminalis from junction of corpus and ductus bursae in the female genitalia (Fig. 10). It is quite similar to *Ectomyelois bipectinalis* sp. n., but with differences as mentioned in the diagnosis of the latter.

#### Distribution.

China (Guangdong, Guangxi, Hainan, Taiwan, Yunnan), Japan, India, Sri Lanka, Sikkim, Israel, Mediterranean, Central Europe, Norway, United Kingdom, North Africa, Australia, Argentina, United States, Puerto Rico, Jamaica.

### Ectomyelois bipectinalis sp. n.

Taxon classificationAnimaliaLepidopteraPyralidae

http://zoobank.org/BF7291BA-0081-45D5-B1F4-6980247E5C59

Figs 3 , 4 , 8 , 11

#### Type material.

**Holotype** ♂, **CHINA**: Guanping (22°14'N, 100°53'E), Xishuangbanna, Yunnan, 1200 m, 19-VIII-2005, coll. Yingdang Ren. **Paratypes**: **Fujian**: 2 ♀♀, Mt. Tianzhu (24°35'N, 117°55'E), 220 m, 8,14-IX-2010, coll. Yinghui Sun & Jing Zhang. **Gansu**: 1 ♂, Fanba (32°44'N, 105°07'E), Wenxian, 718 m, 18-VII-2005, coll. Haili Yu, gen. slide no. RYD04745. **Guangxi**: 1 ♂, Yachang Yard, Leye County (24°47'N, 106°33'E), 665 m, 24-VII-2004, coll. Jiasheng Xu; 1 ♂, Mt. Yuanbao (25°14'N, 109°07'E), 500 m, 10-VIII-2006, coll. Weichun Li, gen. slide no. LYJ10112; 1 ♂, Pingxincun, Yizhou (24°30'N, 108°40'E), 150 m, 16-VIII-2011, coll. Shulian Hao & Yinghui Sun; 1 ♂, 1 ♀, Shaoping Yard, Pingxiang (22°03'N, 106°55'E), 190 m, 24,28-VII-2011, coll. Bingbing Hu; 1 ♀, Qingshan Yard, Pingxiang(22°03'N, 106°55'E), 300 m, 20-VII-2011, coll. Bingbing Hu. **Hainan**: 1 ♂, Mt. Diaoluo (18°39'N, 109°54'E), 70 m, 27-V-2007, coll. Zhiwei Zhang & Weichun Li, gen. slide no. LJY10104; 1 ♀, Mt. Duowen (19°48'N, 109°45'E), 120 m, 2-V-2009, coll. Qin Jin & Bingbing Hu, gen. slide no. LJY10105; 1 ♀, Wuzhishan (18°46'N, 109°30'E), 740 m, 14-IV-2009, coll. Qin Jin & Bingbing Hu, gen. slide no. LJY10097; 1 ♂, Shuimanxiang (18°53'N, 109°40'E), Wuzhishan, 620 m, 19-IV-2014, coll. Tengteng Liu, Wei Guan & Xuemei Hu; 1 ♂, Mt. Limu (19°10'N, 109°44'E), Qiongzhong, 640−700 m, 4-V-2014, coll. Tengteng Liu, Wei Guan & Xuemei Hu; 1 ♂, Wuzhishan (18°40'N, 109°29'E), 500 m, 12-IV-2013, coll. Yingdang Ren & Xiaoguang Liu, gen. slide no. RYD2013046 (deposited in HAASM). **Yunnan**: 4 ♂♂, Rare Botanical Garden, Ruili (24°00'N, 97°50'E), 1000 m, 5−8-VII-2005, leg. Yingdang Ren, gen. slide nos. RYD04718, RYD04718w; 1 ♂, Guanping (22°15'N, 100°53'E), Xishuangbanna, 1200 m, 17-VIII-2005, coll. Yingdang Ren, gen. slide no. RYD04717; 1 ♂, Botanical Garden, Menglun (21°52'N, 101°18'E), 570 m, 13-VIII-2005, coll. Yingdang Ren, gen. slide no. RYD04721; 4 ♂♂, Bubang (21°36'N, 101°35'E), Xisuangbanna, 650 m, 22−24-VIII-2005, coll. Yingdang Ren, gen. slide nos. LHX14081, LHX14081w; 1 ♂, Botanical Garden (21°55'N 101°16'E), Xishuangbanna, 560 m, 1-VIII-2010, coll. Yinghui Sun & Lixia Li; 2 ♂♂, Bakaxiaozhai (21°58'N, 101°12'E), Mengla, Xisuangbanna, 630 m, 7-VIII-2010, coll. Yinghui Sun & Lixia Li; 7 ♂♂, 1 ♀, Mengyuan (21°42'N, 101°23'E), Mengla, Xishuangbanna, 640 m, 10~13-VIII-2010, coll. Yinghui Sun & Lixia Li, gen. slide nos. LJY10351, LJY10352; 20 ♂♂, 16 ♀♀, Bubang (21°36'N, 101°35'E), Mengla, 650 m, 12~14-VII-2013, coll. Linlin Yang, gen. slide nos. RYD20120106m, RYD20120107m, RYD20120167f, RYD20120168m (deposited in HAASM).

#### Diagnosis.

This new species is notable superficially for its bipectinate male flagellum. It is much more similar to *Ectomyelois ceratoniae* in genitalic structures, but can be distinguished from the latter by the narrower uncus with width almost equaling length, the widest part is at basal 2/5, the apical projection of gnathos approximately 3/5 length of uncus in male, and the elongate corpus bursae three times as long as wide in female. In *Ectomyelois ceratoniae*, the uncus is more wide than long, the widest part at base, the apical projection of gnathos nearly the same length as uncus in male, and the corpus bursae is twice as long as wide in female.

#### Description.

Wingspan 19.5−28.0 mm (Figs 3, 4). Vertex brown, with individual scales tipped with grayish white. Antenna (Fig. 3a, b) brown; bipectinate, with pecten about twice length of width of shaft in male, shortly ciliate in female. Labial palpus brown, individual scales white-tipped. Maxillary palpus brown. Occiput, patagium, tegula and thorax brown. Forewing dark grayish fuscous, blackish brown along veins; antemedial line invisible; discal spots blackish brown, separated; postmedial line faint, grayish white, serrated; terminal line black, interrupted; cilia brown. Hindwing grayish white, pale brown along costa, termen and veins; cilia grayish white. Legs brown, mottled with grayish white, spurs grayish white. Abdomen yellowish brown.

**Male genitalia** (Fig. 8). Uncus bell-shaped, width almost equals length, rounded at apex, triangularly protruded laterally at basal 2/5, arched on basal margin. Apical projection of gnathos about 3/5 length of uncus, clubbed, slightly bent and furcated at apex. Transtilla trefoiled, including a pair of triangularly inflated bases, and a tongue-shaped central projection with rounded apex posteriorly. Valva three times as long as wide, evenly curved toward rounded apex, costal margin almost parallel with ventral margin except slightly convex at basal 2/3; costa strongly sclerotized, broad at base, slightly narrowed and extending to end of valva, without process apically; sacculus strongly sclerotized, stout, 2/5 length of valva. Juxta U-shaped, base an arched belt, with a pair of wide, stout, incurved lateral lobes, expanded and bearing sparse setae apically. Vinculum U-shaped, length almost equal to the widest posterior margin, slightly concave at middle of anterior margin. Phallus slightly shorter than valva, with membranous crimples internally; cornutus absent. Eighth tergite fan-shaped, 4/5 length than width, with a pair of spoon-like sclerites anteriorly; eighth sternite with a pair of triangular plates narrowly connected anteriorly. Culcita (sensu Amsel 1956) simple, one pair of fine scale tufts.

**Female genitalia** (Fig. 11). Anal papillae triangular, with a few setae, blunt apically. Eighth tergite slightly concave on posterior margin, trapezoidally convex on anterior margin; eighth sternite with membranous part inverse-funneled. Antrum twice as wide as length. Ductus bursae membranous, twice length of apophyses posteriores. Corpus bursae membranous, elongate, slightly shorter than ductus bursae, three times as long as wide; signum an elongate patch of microspines, placed at posterior 2/5. Ductus seminalis from junction of corpus bursae and ductus bursae.

#### Distribution.

China (Fujian, Gansu, Guangxi, Hainan, Yunnan).

#### Etymology.

The specific name is derived from the Latin prefix *bi*-, meaning two, and the Latin *pectinalis*, meaning pectinate, referring to the bipectinate male flagellum.

### Ectomyelois furvivena sp. n.

Taxon classificationAnimaliaLepidopteraPyralidae

http://zoobank.org/72178A69-8258-4F83-A8C0-D1153B7E86CE

Figs 5 , 6 , 9 , 12

#### Type material.

**Holotype** ♂, **CHINA**: Rare Botanical Garden, Ruili (24°00'N, 97°50'E), Yunnan, 1000 m, 8-VII-2005, leg. Yingdang Ren, gen. slide no. RYD04737. **Paratypes**: **Gansu**: 1 ♂, Fanba (32°44'N, 105°07'E), Wenxian, 718 m, 18-VII-2005, coll. Haili Yu, gen. slide no. RYD04744. **Yunnan**: 2 ♂♂, same data as for holotype, gen. slide nos. LHX14084, LHX14084w, LHX14085; 1 ♀, Botanical Garden, Menglun (21°52'N, 101°18'E), 570 m, 13-VIII-2005, coll. Yingdang Ren, gen. slide no. RYD04720; 1 ♂, Baihualing, Mt. Gaoligong (25°31'N, 98°32'E), 1470 m, 30-VII-2013, coll. Linlin Yang, gen. slide no. RYD20120181 (deposited in HAASM).

#### Diagnosis.

This new species is similar to *Ectomyelois bipectinalis* sp. n., but can be recognized by the male antenna is not bipectinate, the uncus is rather abruptly narrowed beyond its broad base, tapered apical projection of the gnathos is about half length of the uncus and the inverse-goblet transtilla in the male genitaia. In *Ectomyelois bipectinalis* sp. n., the antenna is bipectinate, the uncus protrudes triangularly at basal 2/5, the apical projection of gnathos is about 3/5 length of the uncus and the transtilla is trefoiled in the male genitalia. There is little difference in the female genitalia except the corpus bursae is much broader and the signum is smaller than in *Ectomyelois bipectinalis* sp. n.

#### Description.

Wingspan 25.0−30.0 mm (Figs 5, 6). Vertex brown, with individual scales grayish white-tipped. Antenna (Fig. 5a, b) brown, scales dark-tipped. Labial palpus brown, first segment with scales grayish white-tipped. Maxillary palpus brown. Occiput, patagium, tegula and thorax grayish brown, with scales tipped with grayish white. Forewing dark grayish brown with some white powdering, black along veins; antemedial line invisible; discal spots blackish brown, separated; postmedial line faint, grayish white, serrated, gently curved inwardly from costal 1/5 to dorsum 1/5; terminal line black, interrupted; cilia brown. Hindwing grayish white, light brown along costa and veins; cilia white. Foreleg blackish brown; mid- and hind legs brown with grayish white powdering, spurs yellowish brown. Abdomen with each tergite gray basally and grayish white distally, sternite yellowish brown.

**Male genitalia** (Fig. 9). Uncus bell-shaped, length longer than wide, abruptly narrowed beyond its broad base, rounded at apex. Apical projection of gnathos about half length of uncus, tapered, slightly bent and furcated at apex. Transtilla inverse-goblet shaped; deeply concaved in U shape on anterior margin, a rounded plate protruding on posterior margin. Valva three times as long as wide, evenly curved toward rounded apex, costal margin almost parallel with ventral margin, ventral margin concave at basal 1/3; costa strongly sclerotized, broad at base, narrowed and extending to near end of valva, without process apically; sacculus strongly sclerotized, stout, 2/5 length of valva. Juxta a broad, quadrate plate; lateral lobes ovate, 1.5 times as long as wide, bearing sparse setae in distal half. Vinculum trapezoid, widest posterior margin about 1.6 times of its length, straight on anterior margin. Phallus about 2/3 length of valva, smooth inside; cornutus absent. Eighth tergite cupped, with a pair of triangular sclerites anteriorly; eighth sternite with a pair of boot-like sclerites narrowly connected anteriorly. Culcita simple, one pair of fine scale tufts.

**Female genitalia** (Fig. 12). Anal papillae triangular, with a few setae, blunt apically. Eighth tergite slightly concave on posterior margin, convex W-shaped on anterior margin; eighth sternite with membranous part inverse-funneled. Antrum somewhat quadrate. Ductus bursae membranous, 1.5 times length of apophyses posteriores. Corpus bursae membranous, about same length as ductus bursae, twice as wide; signum a spindle-like patch of scobinations, at middle of corpus bursae. Ductus seminalis from junction of corpus bursae and ductus bursae.

#### Distribution.

China (Gansu, Yunnan).

#### Etymology.

The specific name is derived from the Latin prefix *furv*-, meaning black, and the Latin *vena*, vein, referring to the forewing with black scales along its veins in this species.

## Discussion

The genus *Ectomyelois* is characterized by the wing venation, a signum with a patch of microspines and the inception of the ductus seminalis in the female genitalia. Two new species are assigned to this genus based on these characters. *Ectomyelois bipectinalis* sp. n. is unique for its bipectinate male flagellum, but the other characters, especially the genitalia, are in accord with the generic characters.

Neunzig and Goodson (1992) described one new species, *Ectomyelois austrella* Neunzig & Goodson, 1992, from Argentina. However, the male genitalia has a basal protuberance on the valva not found in *Ectomyelois* species, and the female genitalia bears a narrowly and deeply invaginated signum on the corpus bursae also not found in *Ectomyelois* species. Although we retain this species in *Ectomyelois*, the characters indicate that *austrella* might not be suitably placed in this genus.

## Supplementary Material

XML Treatment for Ectomyelois

XML Treatment for Ectomyelois ceratoniae

XML Treatment for Ectomyelois bipectinalis

XML Treatment for Ectomyelois furvivena
